# Catheter Colonization and Abscess Formation Due to *Staphylococcus epidermidis* with Normal and Small-Colony-Variant Phenotype Is Mouse Strain Dependent

**DOI:** 10.1371/journal.pone.0036602

**Published:** 2012-05-07

**Authors:** Gunnar Sander, Tina Börner, André Kriegeskorte, Christof von Eiff, Karsten Becker, Esther Mahabir

**Affiliations:** 1 Comparative Medicine, Center for Molecular Medicine, University of Cologne, Cologne, Germany; 2 Institute of Medical Microbiology, University Hospital of Münster, Münster, Germany; Duke University Medical Center, United States of America

## Abstract

Coagulase-negative staphylococci (CoNS) form a thick, multilayered biofilm on foreign bodies and are a major cause of nosocomial implant-associated infections. Although foreign body infection models are well-established, limited *in vivo* data are available for CoNS with small-colony-variant (SCV) phenotype described as causative agents in implant-associated infections. Therefore, we investigated the impact of the *Staphylococcus epidermidis* phenotype on colonization of implanted PVC catheters and abscess formation in three different mouse strains. Following introduction of a catheter subcutaneously in each flank of 8- to 12-week-old inbred C57BL/6JCrl (B6J), outbred Crl:CD1(ICR) (CD-1), and inbred BALB/cAnNCrl (BALB/c) male mice, doses of *S. epidermidis* O-47 wild type, its *hemB* mutant with stable SCV phenotype, or its complemented mutant at concentrations of 10^6^ to 10^9^ colony forming units (CFUs) were gently spread onto each catheter. On day 7, mice were sacrificed and the size of the abscesses as well as bacterial colonization was determined. A total of 11,500 CFUs of the complemented mutant adhered to the catheter in BALB/c followed by 9,960 CFUs and 9,900 CFUs from *S. epidermidis* wild type in BALB/c and CD-1, respectively. SCV colonization was highest in CD-1 with 9,500 CFUs, whereas SCVs were not detected in B6J. The minimum dose that led to colonization or abscess formation in all mouse strains was 10^7^ or 10^8^ CFUs of the normal phenotype, respectively. A minimum dose of 10^8^ or 10^9^ CFU of the *hemB* mutant with stable SCV phenotype led to colonization only or abscess formation, respectively. The largest abscesses were detected in BALB/c inoculated with wild type bacteria or SCV (64 mm^2^ vs. 28 mm^2^). Our results indicate that colonization and abscess formation by different phenotypes of *S. epidermidis* in a foreign body infection model is most effective in inbred BALB/c followed by outbred CD-1 and inbred B6J mice.

## Introduction

The opportunistic pathogen *Staphylococcus epidermidis*, member of the group of coagulase-negative staphylococci (CoNS) and usually colonizing the human skin and mucous membranes, is one of the most frequently isolated pathogens involved in nosocomial device-associated infection. Clinical experience with such infections shows that often neither host defense mechanisms nor antibacterial therapy are able to cure these bacteria, probably due to the ability of *S. epidermidis* to form a thick, multi-layered biofilm on surfaces of implanted or inserted foreign bodies [1]. Consequently, when treatment fails, catheters or prostheses have to be replaced.

The bacterial genetic and regulatory factors leading to biofilm formation has been investigated in several studies and the concept of cell-density-dependent quorum sensing has been identified as an important factor for bacterial communication and induction of the formation of biofilm communities. In contrast to *S. aureus*, *S. epidermidis* produces only a limited amount of toxins and degradative exoenzymes. As such, investigations of *S. epidermidis* biofilms and its potential as a virulence factor were intensively performed in animal models in the past three decades, but resulted in conflicting data with respect to biofilm formation. Reports differed depending on the bacterial strain, the presence of foreign bodies such as catheters, tissue damaging caused by insertion or removal of foreign bodies, and the choice of suitable mouse strains [2], [3], [4], [5], [6].

The recovery of small-colony variants (SCVs) of CoNS and their involvement in device-related infection, including pacemaker-related infections, became eminent in the past decade [7], [8], [9]. SCVs are characterized as small-growing subpopulations of bacteria with changed physiological and biochemical traits, often correlated with auxotrophisms for menadione, thymidine and/or hemin [10]. SCVs have been associated with chronic, long-lasting and recurrent infections, and it was suggested that this property was linked to the ability of SCVs to survive intracellularly, thereby being protected from the host immune system [11], [12]. Compared to its normal phenotype counterpart, an augmented expression of polysaccharide intercellular adhesin, the main component of *S. epidermidis* biofilms, was detected in a *hemB* mutant with SCV phenotype [13]. The influence of the bacterial phenotype on biofilm formation, virulence, and on the potential to cause chronic and recurrent infection has not been investigated *in vivo* to date.

In order to elucidate the role of the bacterial phenotype on infection, the importance of a critical infectious dose and the strain of mouse as host used in such studies, we established a bacterial phenotype-, dose-, and host-dependent *S. epidermidis* foreign-body-infection model.

## Materials and Methods

### Bacterial strains, growth conditions, and growth curves

Wild-type *S. epidermidis* O-47 with normal phenotype, its *hemB* mutant with SCV phenotype, and its complemented mutant displaying the wild-type phenotype were grown on tryptic-soy-agar (TSA, Sigma Aldrich, Germany) or in tryptic-soy-broth (TSB, Sigma Aldrich) at 37°C and aerated at 180 rpm [13]. Bacteria from overnight cultures were inoculated 1∶100 in a medium-to-flask ratio of 1∶10 and grown to cell densities appropriate for the bacterial doses required. Antibiotics were purchased from Sigma and were added to the medium at final concentrations of 5 µg/ml erythromycin to the *hemB* and complemented mutant, because of introduced resistance cassettes [13]. The latter was also supplemented with 10 µg/ml chloramphenicol [13]. Growth curves were determined by measuring the optical density at λ = 578 nm over 12 hours. Live-cell determination was performed by plating adequate dilutions of growing cultures on TSA each hour and counting the number of counting colony-forming units (CFU) after at least 24 h of incubation as described previously [14].

### Mice and Husbandry

Inbred C57BL/6JCrl (B6J), outbred Crl:CD1(ICR) (CD-1), and inbred BALB/cAnNCrl (BALB/c) mice were introduced via embryo transfer and bred in a full barrier unit at the CMMC animal facility. Breeding colonies were kept in individually ventilated cages (IVCs, Tecniplast, Italy) at a temperature of 20 to 24°C, humidity of 50 to 60%, 60 air exchanges per hour and a 12/12-hour light/dark cycle. Wood shavings (Ssniff, Germany) were provided as bedding. Mice were fed a standardized mouse diet (1314, Altromin, Germany) and provided drinking water *ad libitum*. All materials, including IVCs, lids, feeders, bottles, bedding, and water were autoclaved before use. Sentinel mice were investigated and monitored negative for all murine infectious agents including *S. epidermidis*. Experimental and control mice were kept in IVCs under negative pressure and the conditions stated above. All animal manipulations were performed in a class II laminar flow biological safety cabinet (Tecniplast).

### Foreign-body-infection model

**Figure 1:**
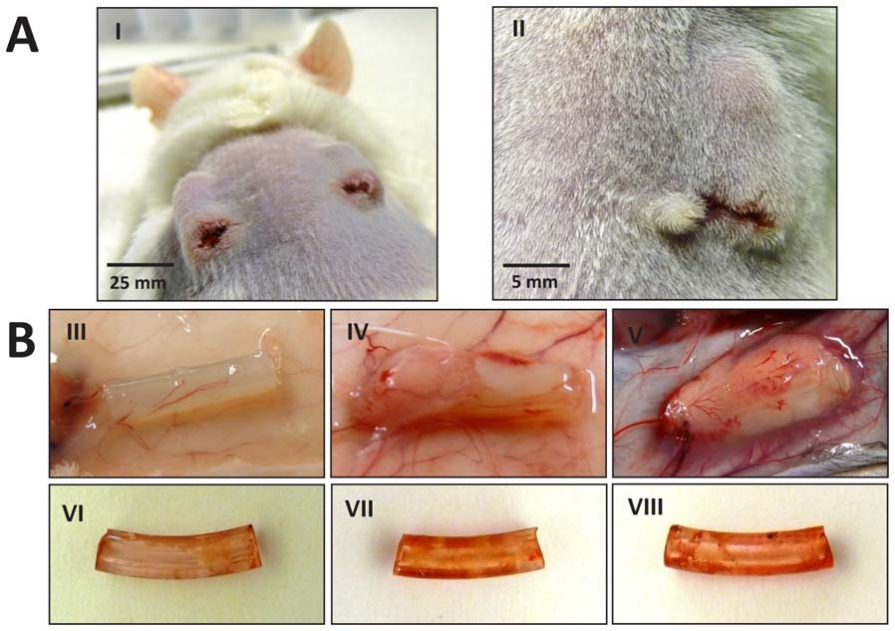
Post-operative view of mice, abscesses, and biofilm staining of catheters. Figure 1A, I: Implantation of a 1-cm long sterile PVC catheter segment subcutaneously subsequent to anesthetizing, shaving, and making a small incision in each flank of the mouse. Figure 1A, II. Abscess formation in a mouse 7 days post inoculation with *S. epidermidis* O-47. Figure 1B: Subcutaneous abscesses from mice 7 days after inoculation of *S. epidermidis* O-47; III) BALB/c mouse with a dose of 10^7^, IV) CD-1 mouse with a dose of 10^8^, and V) B6J mouse with a dose of 10^9^ CFUs and safranin-stained PVC catheters from BALB/c mice 7 days after inoculation of *S. epidermidis* O-47; VI) dose of 10^7^, VII) dose of 10^8^, and VIII) dose of 10^9^ CFUs.

A foreign-body-infection model was performed as described previously with modifications [6]. Briefly, male mice, aging 8 to 12 weeks, were anaesthetized, shaved dorsally, and the skin was disinfected with Cutasept (Bode, Germany). A 0.5-cm incision in each flank was made and two 1-cm long sterile PVC catheter segments were implanted subcutaneously (Figure 1A, I). Doses from PBS-washed overnight cultures of *S. epidermidis* O-47, its *hemB* mutant with stable SCV phenotype, or its complemented mutant at concentrations of 10^6^, 10^7^, 10^8^ or 10^9^ CFUs per 50 µl in 0.9% NaCl were gently spread onto each catheter (one dose per mouse; two catheters per mouse). Wounds were closed with absorbable sutures and wound clips. For each infection dose and bacterial strain, four mice were inoculated (overall 48 mice per strain). Each of additional four negative controls received 50 µl of 0.9% NaCl per catheter. An aliquot of the bacterial suspension used was subsequently streaked out in appropriate dilutions on TSA to confirm doses. On day 7, mice were sacrificed by cervical dislocation and the abscesses were measured (Figure 1A, II; 1B V). To determine the number of adherent bacteria, catheters were removed and washed twice with PBS (n = 7 for groups with bacteria; n = 3 for negative controls). Tween-EDTA buffer was added prior to 3 minutes of sonication and vortexing. The supernatant and adequate dilutions were streaked out on TSA, incubated at 37°C for at least 48 hours and CFUs were counted. A drop of blood, wound (approx. 5 mm×3 mm skin biopsy surrounding incision), and abscess samples were incubated in 10 ml TSB for at least 48 h. Single colonies from positive cultures were isolated on TSA. Identification and confirmation of subcultured bacteria were performed by susceptibility tests for the *hemB* mutant and the complemented mutant, and additionally 16S ribosomal RNA gene sequencing. The biofilm formation on catheters (n = 1 for each dose) was determined by safranin staining as described previously [15], [16], [17]. Briefly, catheters were air-dried overnight, stained in 0.1% safranin for 30 s, air-dried, and the staining intensity was monitored.

### Statistical analysis

Statistical analysis was performed using the unpaired Student *t*-test. Values of p<0.05 were considered as significant.

### Ethic statement

All animal experiments were conducted and approved by local authorities (“Landesamt für Natur, Umwelt und Verbraucherschutz”, North Rhine Westphalia; reference number 87-51.04.2010.A353) in accordance with German law of animal protection (18^th^ of May 2006 (BGBI.I S. 1206 1313) which was amended on the 18^th^ of December 2007 (BGBI I S. 3001; 2008, 47).

## Results

All mice included in this study showed no symptoms of systemic infection during the 7-day-period of the investigation. Wound healing in infected mice was neither affected nor retarded compared to controls. However, mice lost 7% (B6J, CD-1) to 9% (BALB/c) body weight until the end of experiment when infected with *S. epidermidis* normal phenotype in doses of 10^9^ (data not shown, p>0.05).

No abscess formation was observed on day 7 at doses of 10^6^ and 10^7^ CFUs per catheter. Data for abscess formation on day 7 for all three mouse strains at doses of 10^8^ and 10^9^ CFUs of *S. epidermidis* O-47 or the complemented mutant are presented in Table 1. At this dose, abscesses detected from O-47 infection were significantly larger in BALB/c, measuring 22.5 mm^2^, compared to the abscesses from CD-1 and B6J. Figure 1B, III-V shows representative abscess formation with *S. epidermidis* O-47 in the three different mouse strains inoculated with doses of 10^7^–10^9^ bacteria per catheter. Abscess formation was not observed in all mice below a dose of 10^8^ *hemB* mutant bacteria displaying the SCV-phenotype. Starting at a dose of 10^9^, the *hemB* mutant resulted in abscess formation in all mouse strains with sizes being significantly different among all three strains, but smaller compared to abscesses due to *S. epidermidis* O-47. Compared to B6J and CD-1, the largest abscesses were detected in BALB/c with an infectious dose of 10^9^ of the complemented mutant (69 mm^2^, p<0.05).

**Table 1:** Abscess size and colonization of catheters according to dose of three *Staphylococcus epidermidis* strains in inbred C57BL/6JCrl , outbred Crl:CD1(ICR), and inbred BALB/cAnNCrl mice#.

	Abscess size (mm^2^), strain of mice, and bacterial dose (CFUs*)						Colonization of catheters (no. of CFUs ± SEM‡), strain of mice, and bacterial dose (CFUs)					
Bacteria	C57BL/6JCrl		Crl:CD1(ICR)		BALB/cAnNCrl		C57BL/6JCrl		Crl:CD1(ICR)		BALB/cAnNCrl	
	10^8^	10^9^	10^8^	10^9^	10^8^	10^9^	10^8^	10^9^	10^8^	10^9^	10^8^	10^9^
*S. epidermidis* WT§	12±1^a^	41.8±3^a^	10±2.3^a^	39.8±3.5^a^	22.5±5.4^b^	63.8±4.7^a^	286±97^a^	1,235±60^a^	5,993±1,937^a^	9,900±3,580^a^	2,468±1,910^a^	9,960±3,340^a^
*S. epidermidis hemB* £	0^a^	16.5±1^a^	0^a^	7.8±1.1^b^	0^a^	28.3±5.5^c^	56±46^a^	0^a^	1,215±454^a^	9,540±3,659^b^	4,195±3,425^a^	3,305±2,330^a^^,^ b
*S. epidermidis* CM+	27±2.7^a^	26±4^a^	8.75±1.6^a^	55±2.3^b^	19.5±6.5^a^	68.8±5.5^b^	3,917±1,497^a^	3,220±2,161^a^	807±536^a^	404±39^a^	5,800±2,333^a^	11,524±8,168^a^

# four 8–12 week old male mice per bacterial- dose and phenotype were used.

* CFUs: colony-forming unit,

‡ SEM: standard error of the mean,

§ WT: wild type,

£ *hemB*: *hemB* knock-out mutant with small-colony-variant phenotype,

+ CM: complemented mutant of *S. epidermidis hemB*.

a–c Values with different superscripts within a row, assigned to the same infectious dose, and separated between abscess size and colonization vary significantly (p<0.05; Student's unpaired *t*-test).

As shown in Table 1, an inoculation dose of 10^9^ CFUs per catheter resulted in a recovery of 11,524 CFUs of the complemented mutant from BALB/c followed by 9,960 CFUs and 9,900 CFUs of *S. epidermidis* O-47 from BALB/c and CD-1, respectively. Colonization with the *hemB* mutant displaying the SCV phenotype was most effective in CD-1 with 9,540 CFUs at a dose of 10^9^ CFUs per catheter, whereas no *S. epidermidis hemB* mutant bacteria were detected in B6J (p<0.05). The number of bacteria recovered at lower doses of 10^6^ and 10^7^ ranged from a minimum of zero (CD-1 including all three bacterial strains and BALB/c with the *hemB* mutant at a dose of 10^6^ bacteria per catheter) to a maximum colonization of 1,230 CFUs (BALB/c infected with the complemented mutant, see Table S1).

In Figure 1B, VI–VIII, safranin staining of representative catheters is shown. Biofilm formation by bacteria was detected at doses of 10^8^ and 10^9^ bacteria per catheter, whereas none was observed at doses of 10^6^ and 10^7^ (data not shown). With increasing number of adherent bacteria, the red staining became more intensive. Negative controls had the same intensity of staining as shown in Figure 1B, VI.

The expected bacteria were solely confirmed in all samples of abscess and in wound cultures where catheter colonization was observed. All blood cultures remained negative.

## Discussion

Although studies investigating the virulence of *S. epidermidis* in foreign-body-infection models in different mouse hosts have been performed [2], [3], [4], [5], [6], we report for the first time an attenuation of colonization and abscess formation in mice infected with an SCV phenotype of *S. epidermidis* compared to the normal phenotype in three different immunocompetent mouse strains. As it is still unknown whether SCV formation is solely due to bacterial adaption to host or intracellular conditions, and/or is the result of mutations, we performed this model of acute foreign-body-infection for an observation period of seven days. [12], [18], [19], [20].

The results reveal an *S. epidermidis* dose-dependent colonization of catheters in immunocompetent mice. Neither the dose of 10^6^ nor 10^7^ led to significant replication of bacteria in all three mouse strains probably due to the fact that adherence to abiotic surfaces is mainly mediated through biofilm formation and expression of adhesins, which lead to better survival in the host [21]. Quorum sensing, which depends on cell density, might explain colonization of catheters at a minimum dose of 10^8^ bacteria, which seems to be the initial dose required for inducing effective biofilm formation in this model. Compared to doses of 10^6^, which often have been used in *S. aureus* animal models, the high dose of *S. epidermidis* used in our model is probably due to the fact that *S. epidermidis* lacks mass of virulence factors, which are present in *S. aureus*, and its versatile potential to evade host defense mechanisms [22], [23].

As described in several studies for *S. aureus*, the *hemB* mutant with SCV phenotype differs in virulence compared to the normal phenotype depending on the *in vitro* or *in vivo* model used [12], [24], [25]. *S. aureus hemB* mutants have been shown to survive intracellularly and display reduced virulence *in vitro* [12]. Although the *S. epidermidis hemB* mutant is described to produce more biofilm than the normal phenotype *in vitro*, in the present study it does not colonize catheters at amounts comparable to that of the normal phenotype with the exception of CD-1 mice and at a dose of 10^8^ bacteria in BALB/c mice. This is confirmed by the observation that abscesses from *S. epidermidis* displaying the SCV phenotype are smaller than those formed by the normal phenotype. As such, our *in vivo* results do not confirm previous *in vitro* findings [13]. This is most likely due to a combination of defects in electron transport, resulting in growth retardation which *in vivo* leads to reduced biofilm formation and might also lead in consequence to a reduced survival in the mouse. A possible intracellular survival of the *S. epidermidis hemB* mutant, as shown for the *S. aureus hemB* mutant, is not excluded but needs further studies [11], [25]. However, standardized *in vitro* conditions differ not only in nutrition availability, oxygen- and salt concentration, pH, but also in the influence and attack of the immune system to growing bacteria *in vivo*. Thus, the difference between *in vitro* and *in vivo* findings is not quite surprising.

The observation that the complemented mutant does not behave exactly as *S. epidermidis* O-47 (see Table 1) in terms of catheter colonization and abscess formation may be due to the fitness-related fact that this mutant expresses two additional resistance genes, namely, erythromycin and chloramphenicol.

In general, all mice showed no symptoms of systemic infection. Bacteria were not detected in blood indicating that mice were infected only locally. We can not exclude that a possible dissemination to other tissues or blood might occur afterwards. It is possible that a critical cell density in the biofilm community leads to detachment and spreading of bacteria which was not achieved during 7 days of experiment [26], [27], [28]. In addition, the area of the skin in the periphery of the abscess did not show any pathological lesions, and wound healing was not delayed.

Our results indicate that colonization of catheters and abscess formation by different phenotypes of *S. epidermidis* in a subcutaneous foreign-body-infection model is most effective in inbred BALB/c followed by outbred CD-1 and inbred B6J mice. Different results with immunocompetent strains of mice have been reported previously [2], [3], [4], [5], [6], [29] but the underlying molecular mechanisms are mostly unknown. Nevertheless, the different potential of *S. epidermidis* strains to produce biofilm and the different methods for determination of colonization also contribute to the differences in results obtained by different authors [2], [3], [4], [5], [6]. Furthermore, the route and site of infection in addition to host-dependent factors play a major role [29], [30], [31]. In a subcutaneous infection model without a foreign body where 2×10^7^ CFUs of *S. aureus* SH1000 were inoculated in the left hind footpad of 8–12-week old mice, Nippe et al. showed that, in contrast to our findings, B6J mice were more susceptible to *S. aureus* than BALB/c mice, most probably due to strain-dependent differences in granulocyte recruitment during infection [29]. Granulocyte recruitment and *S. epidermidis* biofilm formation may be the reason for faster abscess formation in BALB/c mice than in B6J mice in our study (see Table 1). Surrounded by the abscess the bacteria might be protected from other immune components and may therefore have a better survival chance compared to the situation in B6J at least during the seven day observation period. Thus, when immunocompetent strains are needed for subcutaneous foreign-body-infection models, we recommend using BALB/c mice at a dose of at least 10^9^ *S. epidermidis* O-47 per catheter.

In conclusion, the present study provides further information on choice of mouse strain with regard to bacterial phenotype variants in an *S. epidermidis* infection model. An elucidation of molecular differences *in vivo* between normal and SCV phenotype, immune modulation of the host during infection, and differences between the susceptibility in different mouse strains will be helpful in future investigations involving bacterial and host factors in establishing infections.

## Supporting Information

Table S1 Colonization of catheters according to dose of three *Staphylococcus epidermidis* strains in inbred C57BL/6JCrl, outbred Crl:CD1(ICR), and inbred BALB/cAnNCrl mice
(DOC)
