# Supplementary material for: Catheter Colonization and Abscess Formation Due to Staphylococcus epidermidis with Normal and Small-Colony-Variant Phenotype Is Mouse Strain Dependent
Source: PLoS One. 2012 May 7;7(5):e36602. doi: 10.1371/journal.pone.0036602 (PMC3346766; doi:10.1371/journal.pone.0036602)
Supplement: Table S1 — Colonization of catheters according to dose of three Staphylococcus epidermidis strains in inbred C57BL/6JCrl, outbred Crl:CD1(ICR), and inbred BALB/cAnNCrl mice (DOC) [file pone.0036602.s001.doc]

Table S1

|  | Colonization of catheters (no. of CFUs ± SEM^#^), strain of mice~, and bacterial dose (CFUs*) | | | | | | | |
| --- | --- | --- | --- | --- | --- | --- | --- | --- |
| Bacteria | C57BL/6JCrl | |  | Crl:CD1(ICR) | |  | BALB/cAnNCrl | |
|  | 10^6^ | 10^7^ |  | 10^6^ | 10^7^ |  | 10^6^ | 10^7^ |
| *S. epidermidis* WT^§^ | 2±2.1^a^ | 377±307^a^ |  | 0^a^ | 94±36^a^ |  | 205±97^a,b^ | 231±159^a^ |
| *S. epidermidis hemB^£^* | 153±124^a^ | 130±58^a^ |  | 0^a^ | 80±56^a^ |  | 0^a^ | 5±4^a^ |
| *S. epidermidis* CM^+^ | 43±35^a^ | 707±606^a^ |  | 0^a^ | 25±21^a^ |  | 41±24^a^ | 1,230±886^a^ |

~four 8-12 week old male mice per bacterial- dose and phenotype were used

* CFUs: colony-forming unit, ^#^ SEM: standard error of the mean, ^§^ WT: wild type, *^£^ hemB*: *hemB* knock-out mutant with small-colony-variant phenotype, ^+^ CM: complemented mutant of *S. epidermidis hemB*

^a-b^ Values with different superscripts within a row, assigned to the same infectious dose vary significantly (p<0.05; Student’s unpaired *t*-test
